# Valued personality traits in livestock herding Kelpies—Development and application of a livestock herding dog assessment form

**DOI:** 10.1371/journal.pone.0267266

**Published:** 2022-04-26

**Authors:** Bethany J. Wilson, Elizabeth R. Arnott, Jonathan B. Early, Claire M. Wade, Paul D. McGreevy

**Affiliations:** 1 Sydney School of Veterinary Science, Faculty of Science, University of Sydney, Sydney, New South Wales, Australia; 2 School of Life and Environmental Sciences, Faculty of Science, University of Sydney, Sydney, New South Wales, Australia; 3 School of Environmental and Rural Science, Faculty of Science, University of New England, Armidale, New South Wales, Australia; Memorial University of Newfoundland, CANADA

## Abstract

Livestock herding dogs contribute greatly to the rural economy of Australia. However, their selection currently lacks a cohesive or methodical approach. For example, there is no accessible tool for assessing Australian livestock herding dogs’ suitability for work. The purpose of the current study was to devise a herding dog assessment form, the Herding Dog Assessment Form–Personality (HDAF-P), to facilitate collection of data on relevant behavioural phenotypes of large numbers of working Kelpies and to apply the HDAF-P to identify personality traits needed for herding dog performance. The focus was on creating a succinct form that was salient and accessible to livestock herding dog owners. Wherever practical, terms and methods from published personality questionnaires were integrated. Seventeen terms were included as behavioural descriptors in the HDAF-P which was then used by 95 owners to assess a sample of 228 of their working Kelpies. Owners were also asked to rate the overall ability of their dog(s). Of these dogs, 210 (all twelve months or older) were fully described and their data were used in the analysis. Thus, the study was designed to reveal which personality traits are most critical to the overall ability of the herding dogs and to undertake an exploratory analysis of the patterns of dog behaviour revealed by the HDAF-P in non-juvenile dogs. The traits that showed the strongest correlations (using Kendall’s Tau correlation analysis) with overall ability were initiative (T = 0.41, p < 0.001), persistence (T = 0.36, p < 0.001), intelligence (T = 0.32, p < 0.001), confidence (T = 0.36, p < 0.001) and nervousness (T = -0.30, p < 0.001). An exploratory principal component analysis of trait scores revealed that 64.5% of the variance could be explained by four components that share several similarities with those reported by previous dog personality studies. These findings confirm that the HDAF-P has potential for the practical assessment of livestock herding dog personality and can elucidate traits that should be considered for prioritisation in training and breeding to optimise herding dog ability.

## Introduction

Many farmers rely on livestock herding dogs to herd sheep, cattle and goats in commercial livestock operations, and many participate in competitive livestock herding dog trials [1]. The behaviour and cognition of these dogs are paramount to their success in these endeavours [2].

A hierarchical framework for understanding dog personality has been proposed [3, 4]. In essence, it suggests that personality can be approached at a series of levels whereby ‘super-traits’ are used to explain covariance among groups of personality traits and that those personality traits are a set of factors under which a series of behavioural tendencies cluster.

While, ultimately, it is the behaviour the livestock herding dogs display that matters to their handlers, interrogating all important behaviours in all scenarios is not possible for handlers aiming to select a suitable dog for work or breeding. Therefore, there is merit in identifying an indicative collection of behavioural tendencies that reflect a super-trait continuum in dogs. Once this is achieved, a more granular examination of behavioural tendencies may permit assessment of the personality of dogs that may infer how the animal will deal with future tasks and circumstances.

The benefits of defining and measuring personality in livestock herding dogs include the opportunity to improve the general herding dog population’s performance and longevity in the work-place; in essence, the dogs’ success. Unsuitable personality and behaviour are the major reasons that herding and other working dogs fail [2, 5]. Therefore, identifying the behaviours most relevant to success and measuring these in individual dogs will assist in: placing dogs in appropriate work environments (e.g. sheep or cattle work, yard work or mustering); pairing dogs with appropriate handlers (handlers have different preferences for certain personality types in dogs they work with) and; selecting breeding stock to reach specific breeding goals efficiently.

Conventional methods of selecting dogs for breeding are likely to result in suboptimal efficiency because personality assessments in pups have low predictive validity for eventual performance [6–8] and because complex quantitative traits (such as personality traits) are multifactorial. Complex traits arise from the interaction of multiple genes and non-genetic influences, such as maternal behaviour and the ways individual dog have been managed and handled [9]. The selection of animals for breeding is best undertaken with this complexity in mind. The use of estimated breeding values (EBVs) for complex traits improves the rate of genetic gain within breeding programs because EBVs better reflect an animal’s genetic merit for a trait by statistically accounting for non-genetic influences that can be identified [10, 11]. Therefore, generating objective measurements of behavioural traits in livestock herding dogs will provide data that facilitate genetic comparisons between dogs and form the basis of a more sophisticated breeding program than is currently available within the industry.

The challenges of measuring personality in dogs are considerable. For example, a lack of standardised terminology and inconsistent testing protocols between studies are two of the issues complicating this endeavour [12, 13]. Nevertheless, multiple approaches have been devised to tackle the challenge of differentiating dogs according to their individual personalities. Broadly, these approaches involve behavioural testing (offering standardised challenges that allow observed behaviour to be coded), behavioural observation (with behavioural ratings assigned) and subjective owner ratings [12, 14]. Questionnaires provide a method for collecting owners’ subjective ratings of their dogs’ personality. Their simplicity and affordability have great appeal, along with the ease with which they can be administered. They also have the advantage of basing assessments over an extended period of observation in varied circumstances rather than a limited number of observations under standardised circumstances (as is required in behaviour testing and observation scenarios). Evidence suggests that, despite their reliance on subjective assessments, owner/handler opinions can be comparable, if not superior, to the results of behavioural testing [15–17].

Considerable effort has already been made to identify optimal traits in working dogs and how to test for them [18–21]. A large data-set is required for the future estimation of breeding values for behaviour traits. To obtain such data, owner questionnaires to measure livestock herding dog personality offer the most practical approach. Potential participants are likely to complete a survey if “they perceive that the questions are salient and if the survey does not impose too much of a load on their time” [22]. Therefore, the design imperatives of the current herding dog personality assessment form prioritised brevity, relevance/salience and accessibility to the target audience.

Preliminary indications of the behaviour traits of relevance to livestock herding dogs have been published in an analysis of eight livestock herding dog manuals [23]. That study reported the frequency of use of 76 terms that described herding manoeuvres, skills and general personality traits. Although the frequency of term use in the manuals provides an indication of the significance of the trait to herding dog performance, the lack of contextual analysis of that study cannot confirm whether particular traits contribute positively or negatively to herding dog outcomes.

Livestock herding dog behaviour traits are often assigned to two separate, but equally important, groups: herding-specific behaviours (such as cast, force, cover, eye, balance) that will be referred here to as ‘working behaviours’ and personality traits that can be observed in both herding and non-herding dogs (e.g., boldness, calmness and sociability) [24–26]. The traits in the latter group are the focus of the current study, drawing as it does from the personality literature on companion dogs and working dogs in non-herding contexts [10, 27, 28]. Because of the specific demands of livestock herding work, detailed examination of the working behaviours required a separate approach that will be addressed elsewhere.

Although the HDAF-P was aligned, wherever possible, with published canine personality questionnaires [10, 27, 28], many such questionnaires were developed primarily for assessment of companion dogs and, as such, are of limited merit in the measurement of behaviour and personality of value in the context of herding livestock. Fundamentally, livestock dogs on farms tend to be housed, trained and engaged with very differently to companion dogs [2]. So, handlers and owners may not observe their herding dogs in contexts that companion dog questionnaires assume to be relevant. Furthermore, rather than attempting to define the full spectrum of dog personality, the current study focuses on identifying and measuring the personality traits of greatest relevance and interest to herding dog handlers and breeders.

The Canine Behavioural Assessment and Research Questionnaire (CBARQ) [29] is a widely adopted tool for measuring behavioural traits that has facilitated cross-study comparisons [13, 20, 30]. It has been tested for reliability and validity on large numbers of dogs [29] and now has data on over 130,000 dogs. The questionnaire consists of 100 questions, within 11 subscales, describing dogs’ reactions to certain ‘events, situations and stimuli in their environment’. Most of the subscales examine scenarios considered common for companion dogs (e.g., doorbells, play, access to visitors, fetching). So, it is worth noting that many of these could not be expected to occur with any frequency in a livestock herding dog’s life.

In contrast, the Herding Trait Characterisation (HTC) was developed by the Swedish Sheepdog Society specifically to score relevant personality and herding behaviour in Border collies [10]. Rather than an owner questionnaire, the HTC was designed to be completed by instructors at introductory herding schools after the completion of approximately 7–10 classes. In Australia, no such training and assessment infrastructure exists, so there is no opportunity for the widespread application of a non-competitive, partially standardised HTC testing procedure. However, of the 19 traits measured in the HTC (version 1), five are personality traits (affability towards humans, social behaviour towards dogs, trainability with livestock, trainability without livestock, ability to relax). These may give an indication of the aspects of temperament that are considered important in livestock herding dogs.

The Monash Canine Personality Questionnaire–Revised (MCPQ-R) [27] is an example of another instrument for gathering owner assessments of their dogs’ personality traits. It was developed using a methodology established in the field of human psychology, with a sample size of 1,016 dogs representing all seven Australian National Kennel Council (ANKC) breed groups [31]. It has good reliability, as indicated by test-retest and inter-rater reliability assessments [32]. Results of the questionnaire appear largely to be unaffected by owner and dog demographics and signalment [27].

Although, as an adjective-based questionnaire, the MCPQ-R can be criticised for lacking detailed behavioural information, its style of assessment fulfils the current requirements for simplicity, brevity and relevance. The use of single adjectives can be applied much more easily to the herding dog context than lengthier statements commonly used in other working and companion dog questionnaires e.g., acuity of sense of smell, motivation to retain possession of an object [15] or, begs persistently for food when people are eating [29]. For the current purposes, the MCPQ-R is useful in suggesting patterns of behaviour, many of which are needed for the livestock herding dogs to perform their job.

Impulsivity is described as the inability to delay gratification and to inhibit pre-potent responses [33]. Although not a term used by herding dog owners, nor one that is included in the MCPQ-R, impulsivity is of interest as a potential super-trait or behavioural modifier [16] related to overall success [33]. The Dog Impulsivity Assessment Scale (DIAS) is an 18-item owner-report assessment of impulsive behaviour in dogs. It has convergent validity with behavioural and physiological measures of the impulsivity trait [34].

Impulsivity may have merit in herding dogs so measuring it may be important if it is inversely associated with working success. To work successfully with humans, herding dogs are regularly required to control their behavioural responses, often despite strong competing motivations. Examples include responding to commands to stop and stay (despite a strong motivation to herd stock); herding stock slowly and quietly (despite excitement and high levels of energy, notably after long periods of confinement); complying with commands (even though previous training may have been inconsistent and confusing) [35] and; tolerating housing typified by social and spatial restriction [2]. Given these requirements, it is hypothesised that, *inter alia*, an ability to cope with frustration is related to success in herding dogs. The current study gathered data to test this hypothesis and to reveal how specific behavioural traits correlate with overall ability.

## Materials and methods

### Ethics statement

Approval for this study was granted from The University of Sydney Human Research Ethics Committee (Approval number 15474). Participation in the survey was taken as informed consent.

### Formation of the HDAF—Personality

#### Term selection

Question items were sourced to refer to behavioural traits of relevance to livestock herding dog owners. Where possible, items were sourced from the MCPQ-R [27], the HTC [10] and the DIAS [28]. However, to select personality traits relevant to working dog breeders and handlers and to conform to the extant stakeholder terminology, primacy was given to behavioural terms commonly used in the herding dog literature, as reported in the livestock working dog literature.

A total of 17 terms were included in the HDAF-P to describe dogs’ behaviour and personality. Twelve of the terms were selected from an analysis of the most frequently referenced personality-related terms in livestock herding dog popular literature [23]. Six of the twelve terms selected from the herding dog literature were shared by the MCPQ-R personality assessment tool. An additional three terms were selected from the MCPQ-R. Two were included to reflect the trait of ‘affability to humans’ explored in the HTC.

A further two terms were lifted from the DIAS to assess the impulsivity of the herding dogs: impulsivity (with the accompanying definition, for consistency with the DIAS) and patience. The personality traits assessed in the HTC were cross-checked for inclusion in HDAF-P, albeit using terminology to fit the Australian working dog context. Table 1 summarises the origin of the 17 terms included in HDAF-P and lists the traits used in the HTC to assess related behaviours.

**Table 1:** The origin of 17 terms included in Herding Dog Assessment Form–personality (HDAF-P). A shaded cell indicates the origin of the HDAF-P term. Traits included in the HTC are listed next to related HDAF-P terms. Note that impulsivity is defined in dogs with sudden, strong urges to act; acting without forethought; acting without considering effects of actions).

	Origin of term			
HDAF-P term	Working dog literature popular terms analysis^a^	MCPQ-R^b^	DIAS^c^	HTC^d^
Intelligence				
Trainability				
Timidness				
Excitability				Ability to relax
Obedience				Trainability
Nervousness				Courage
Boldness				Courage
Initiative taking				
Confidence				Courage
Calmness				Ability to relax
Stamina				
Persistent				Work ethic
Hyperactivity				Ability to relax
Sociability				Affability
Friendliness				Affability
Patience				Ability to relax
Impulsiveness ^e^				

^a^[23]

^b^[27]

^c^[28]

^d^[10]

^e^ The DIAS Overall Questionnaire Score (calculated from the 18 questions) had a strong and significant positive correlation (r = 0.7, p < 0.001) with the single question asking owners to rate their dogs’ impulsiveness against the definition provided [28].

The 18-item herding dog personality assessment form can be viewed at S1 File. The formation of this personality questionnaire is described in detail in the above materials and methods section. The HDAF-P score data for the 228 Kelpies (210 complete) are in the S2 File.

#### Scoring system

All terms were scored on a 5-point scale from ‘very low’ (score 1) to ‘very high’ (score 5) with intermediate options ‘low’ (2), ‘average’ (3) and ‘high’ (4). A sixth option of ‘I don’t know’ was also provided to avoid forcing owners to make judgements they did not feel were well informed.

In recognition of the contextual nature of behaviour, participants were primarily asked to provide scores for dogs’ behaviour in the presence of stock. The terms ‘sociability’ and ‘friendliness’ towards people were the only two traits to be scored by handlers by considering their dogs in non-herding situations.

#### Additional data collection

In addition to rating each dog’s personality profile, participants were asked to score the dog’s overall ability. This was scored on a 5-point scale with the ratings: ‘one of the worst dogs I have ever seen/trained’ (1), ‘below average’ (2), ‘about average’ (3), ‘above average’ (4) and ‘one of the best dogs I have ever seen/trained’ (5). The phrasing of this question was sourced from a published method for assessing the overall ability of trainee search dogs [15].

Several other details were collected for inclusion as fixed and variable effects in subsequent analyses. These included: dog’s age, sex, neuter status, registration number, pedigree, and work environment (yard, droving, paddock, or utility).

#### The subjects

The study included only dogs reported to be purebred Kelpies (n = 228) working within Australia. The participants were recruited through advertising in a series of on-line and printed working dog materials. The assessment form was made available as an on-line questionnaire (Google© Forms). A sample of working Kelpie owners were also approached directly by researchers through the Working Kelpie Council of Australia (WKCA) to participate and did so using a hard copy version of the form. Scores for puppies under 12 months of age (n = 36 of 264 preliminary responses) were separated for subsequent analysis.

Working Kelpie (>12 months of age) owners (n = 95) completed the HDAF-P for 228 individual working Kelpies. Of this sample, 62 Kelpies were reported on by five owners whose participation was directly solicited through the WKCA and who completed the hard-copy form. The remaining majority of respondents provided information by completing the online form. Respondents reported on were males (n = 113, of which 9 were neutered) and females (n = 115, of which 16 were neutered). The Kelpies described ranged from 1 to 14 years of age (Median 4 years).

#### Statistical analysis

Analysis was undertaken using the statistical program R [36]. Assessment form rating scores were converted to a continuous scale with a normal distribution by replacing ordinal score classes with a mean z-value [37, 38].

Phenotypic correlations between all 18 terms (17 personality terms + overall ability) were estimated using Kendal’s Tau as this test does not rely on assumptions about the distribution of the data. The [Kendall] package [39] Kendall() function was employed. This function calculates Kendall’s Tau in a pairwise way so that data could be included from any score sheet with incomplete records (notably where owners selected ‘I don’t know’ for a trait term). Correlations of Tau > 0.3 and < -0.3 with p < 0.01 were considered significant moderate correlation and Tau > 0.5 and < -0.5 were considered strong correlations.

A Kaiser-Meyer-Olkin test was run on the correlation matrix using the KMO() function in the [psych] package [40]. A value of 0.77 suggested adequacy of the data for principal component analysis (PCA) which has merit in this form of behavioural study [41].

An exploratory PCA was undertaken to explore patterns of behaviour in the sample of herding dogs. Four components were selected on the basis of parallel analysis using the [psych] package and examination of a scree plot using the [factoextra] package [42]. As a measure of internal consistency, Cronbach’s alpha was calculated for the terms with loadings over 0.3 in each component.

### Association of owner-awarded personality scores on the HDAF-P to owner impression of overall ability

Dogs were assessed by their owners for the following 17 metrics: confidence; calmness; intelligence; trainability; boldness; patience; timidness; persistence; hyperactivity; initiative; excitability; obedience; nervousness; impulsiveness; sociability; friendliness; and stamina.

Of the 228 dogs twelve months of age or older, 210 had complete data for these 17 metrics (some answers such as “I don’t know”, or invalid responses were removed). Owners assessed the metrics on an ordinal scale [very low | low | average | high | very high]. As principal component analysis required the application of numerical values to these ranks, a normally distributed unobserved latent variable was assumed to underly each metric. The normal distribution of this latent variable was then proportionally truncated, using proportions derived from the owner scores, and the mean of each truncated distribution was taken as the numerical value representing that rank [37, 38].

A normally distributed underlying variable (with a mean = 0 and an S.D. = 1) was truncated according to these proportions. The mean of each truncated section was then calculated and this z-value was then assigned as the numerical value for that rank.

### Principal component analysis

A Principal Component Analysis was performed using the stats package of R.

### Mixed model regression of components against overall ability—controlled for other fixed and random effects

In addition to the four principal components listed above, sex, gonad removal, age, and work environment (paddock, yard, utility and droving) were all considered as fixed effects. Owner was considered as a random effect.

Model building consisted of an initial round of univariable fixed effect testing. While overfitting on the final model is a potential concern, the priority in this round was to avoid rejecting potentially explanatory variables. In multivariate modelling, terms were removed by manual stepwise deletion.

## Results

### HDAF-P scores

Table 2 summarises the assessment form results for 228 stock herding Kelpies. For each trait, a high range of scores was reported. There were Kelpies who were scored from 1 to 5 for all traits except for the traits persistence and stamina which had a minimum score given of 2 and a maximum of 5. However, the data were not normally distributed as scores reflected a sample of dogs that tended towards more favourable behaviour, with the data skewed accordingly. The personality trait scores ranged from 1.98 to 4.14 (See Table 2).

**Table 2:** Personality trait scores for 228 working Kelpies scored on a scale from 1 to 5 by owners using the HDAF-P, the number of dogs for which a score was given and the mean and standard deviation of scores given for each term.

	n	Mean	SD
Confidence	228	4.14	0.86
Calmness	228	3.63	1.07
Intelligence	228	4.12	0.83
Trainability	227	3.90	0.88
Boldness	227	3.81	0.95
Patience	227	3.49	0.99
Timidness	228	2.01	0.93
Persistence	228	3.98	0.92
Hyperactivity	228	2.78	1.24
Initiative	226	3.85	0.89
Excitability	227	3.17	1.14
Obedience	228	3.74	0.91
Nervousness	226	1.98	0.95
Impulsiveness	225	2.58	1.13
Sociability	225	4.04	0.98
Friendliness	223	4.33	0.84
Stamina	224	3.99	0.83
Overall ability	184	3.85	0.84

**Fig 1:**
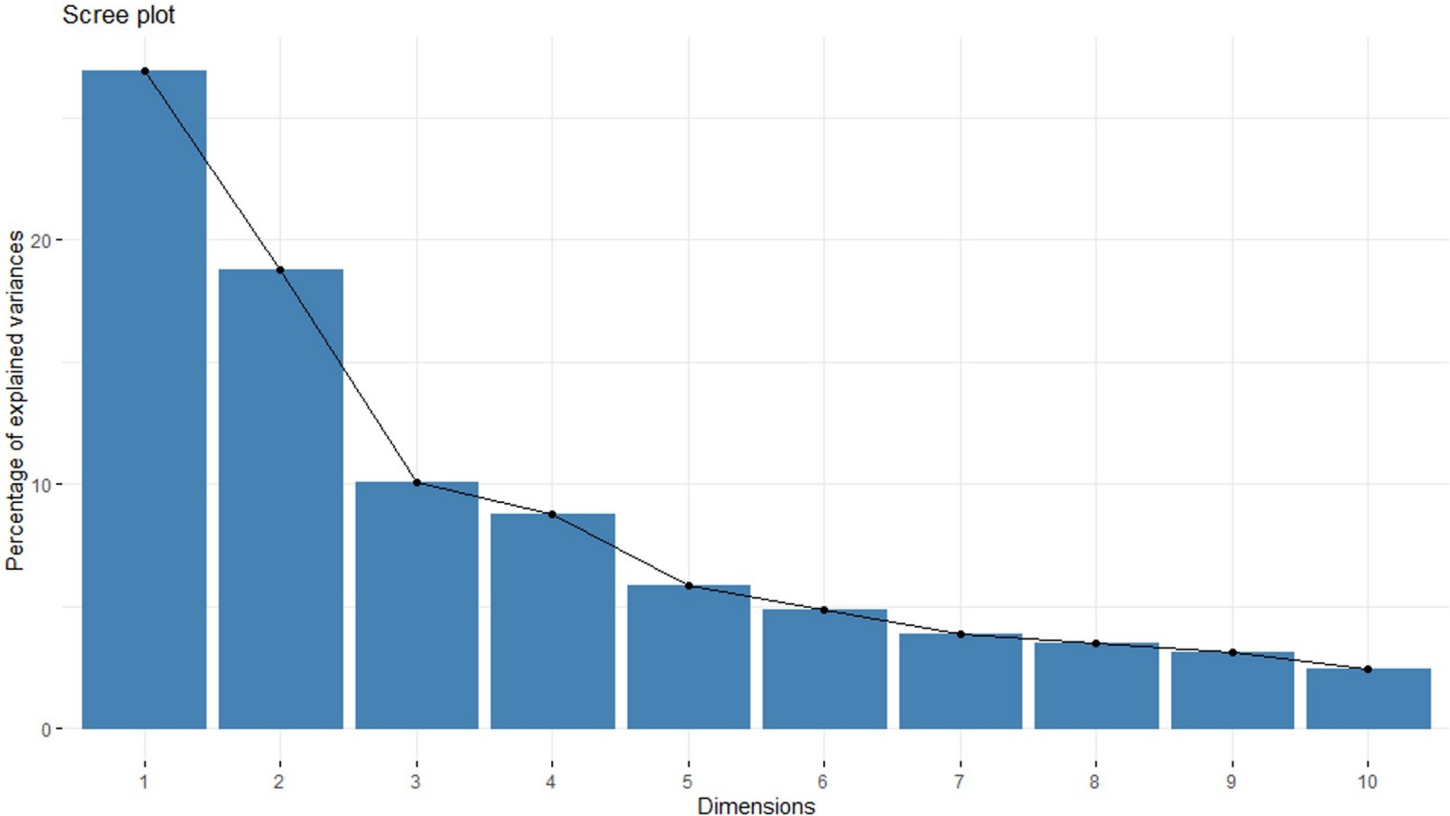
Scree plot of the proportion of variance explained in a principal component analysis working dog personality metrics against the corresponding components. The inflexion point suggests components beyond 3–4 would add relatively little information.

A Scree Plot was generated using package factoextra [42] (Fig 1). As expected, the first few principal components express a high proportion of the total variance (the first four explain approximately 64.5% of the total variance). The partitioning of variance among the components is shown in Table 3.

**Table 3:** Standard deviation, proportion of variance and cumulative proportion of variance of components (c1-c17) from a principal component analysis of working dog personality metrics.

	Component(s)				
	1	2	3	4	5–17
Standard deviation	1.98	1.66	1.21	1.13	0.39–0.92
Proportion of Variance	0.27	0.19	0.10	0.09	0.01–0.06
Cumulative Proportion	0.27	0.46	0.56	0.65	0.35

Maximum likelihood parallel analysis using the psych package [36] and the generated scree plot suggested four components (See Table 4).

**Table 4:** Summary of the results of principal component analyses of owner ratings of 210 working Kelpies on 17 personality terms. Red and orange shading has been used for negative loadings and green and yellow shading for positive loadings with the darker shading indicative of a loading of higher magnitude (> 0.2) than the lighter shading.

	Component			
	1	2	3	4
Confidence	0.228	0.336	0.084	0.004
Calmness	0.34	-0.226	-0.005	0.017
Intelligence	0.34	0.041	-0.172	-0.074
Trainability	0.288	-0.041	-0.39	-0.074
Boldness	0.187	0.401	0.052	0.024
Patience	0.32	-0.25	-0.101	-0.021
Timidness	-0.145	-0.297	-0.38	0.053
Persistence	0.22	0.304	0.064	-0.199
Hyperactivity	-0.245	0.343	-0.355	-0.02
Initiative taking	0.274	0.175	-0.118	-0.249
Excitability	-0.208	0.328	-0.425	-0.02
Obedience	0.252	-0.13	-0.439	-0.005
Nervousness	-0.238	-0.123	-0.347	-0.1
Impulsiveness	-0.262	0.25	-0.054	-0.012
Sociability	0.117	0.143	-0.045	0.668
Friendliness	0.149	0.119	-0.11	0.597
Stamina	0.151	0.21	-0.006	-0.264
**Standard deviation**	**1.98**	**1.66**	**1.21**	**1.13**
**Proportion of Variance**	**0.27**	**0.19**	**0.10**	**0.09**
**Cumulative Proportion**	**0.27**	**0.46**	**0.56**	**0.65**

**Fig 2:**
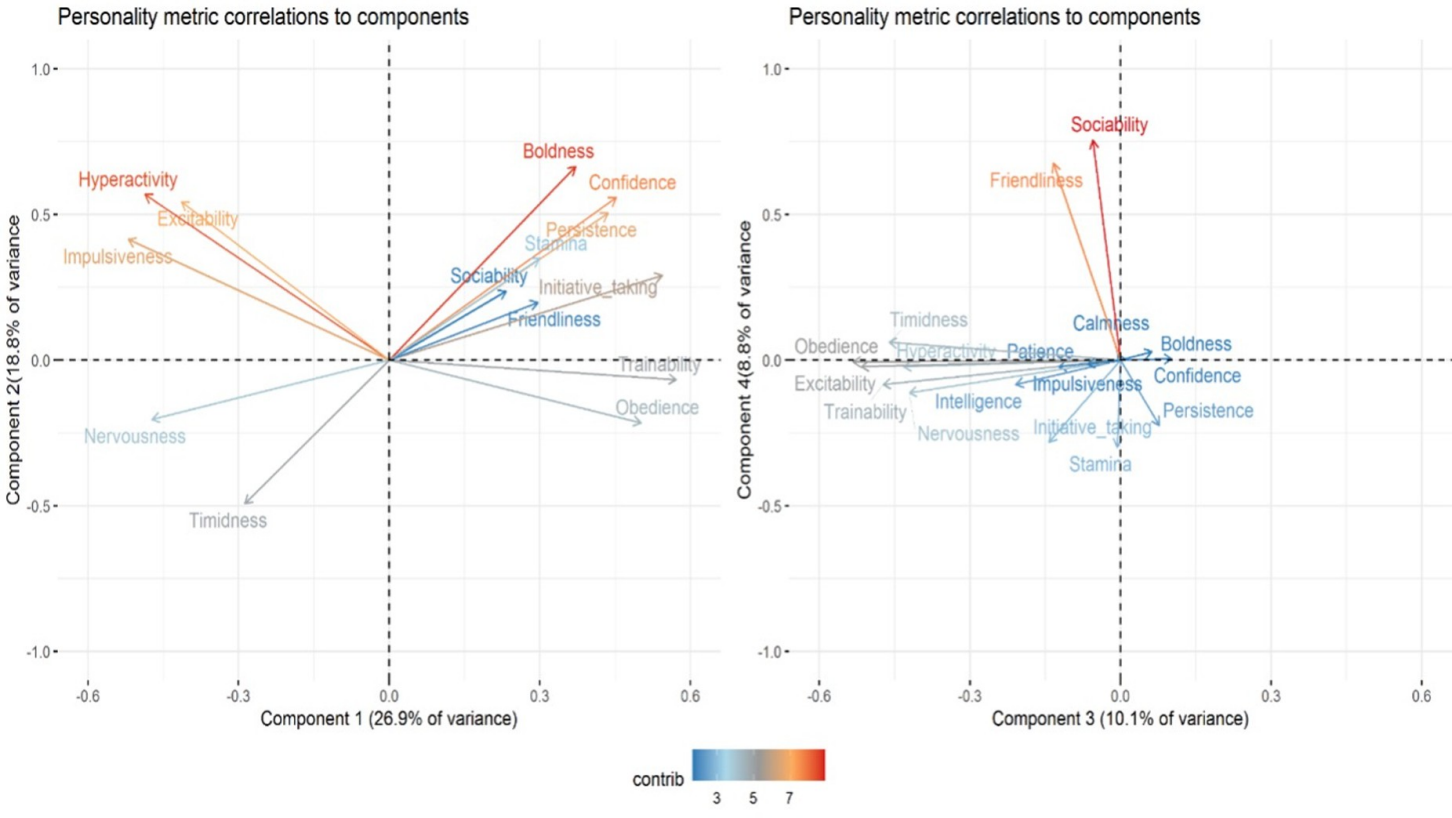
Personality metric correlations to components as revealed by the PCA. a. Component 1 and Component 2. b. Component 3 and Component 4.

The results of the PCA of the ratings for the 210 working Kelpies are summarised in Table 5 and illustrated in Fig 2. The first four components explained approximately 64.5% of the variance. The first component accounted for approximately 26.9% of the behavioural variance. This component supported the internal consistency of the HDAF-P because traits expected to be desirable had converged together (with negative loadings) and diverged from the less desirable traits which, again, were grouped together (with positive loadings). If the focus is on only traits with moderately strong loadings (> 0.3 in magnitude) and terms attributed to the component on which they have the strongest loading, then Component 1 is characterised by behavioural regulation and cognitive ability (calmness, intelligence, patience); Component 2 by a combination of the bold/shy axis and motor activity (boldness, confidence, hyperactivity); Component 3 by trainability and the bold/shy axis (trainability, obedience, timidness, nervousness, excitability) and; Component 4 sociability (sociability and friendliness).

**Table 5:** Results of univariate and final multivariate modelling of relationships between personality traits in four clusters (PC1-PC4) and perceived overall ability in kelpies (n = 210) 12 months of age and older.

	Univariable			Final Multivariable		
	Estimate	T value	P value	Estimate	T value	P value
PC1	0.227	8.242	<0.001	0.213	8.039	0.000
PC2	0.087	2.492	0.013	0.092	3.255	0.001
PC3	0.051	0.965	0.335	0.067	1.597	0.110
PC4	-0.115	-2.068	0.039	-0.087	-1.919	0.055
Sex (male)	0.148	1.217	0.224	0.056	0.574	0.566
Gonads removed	-0.098	-0.534	0.593			-
Age (per year)	0.062	3.058	0.002			
Paddock	0.097	0.707	0.480			-
Yard	-0.011	-0.072	0.942			-
Utility	0.253	1.939	0.053	0.155	1.471	0.141
Droving	0.809	3.944	0.000	0.374	2.131	0.033

### Mixed model regression of components against overall ability—controlled for other fixed and random effects

**Fig 3:**
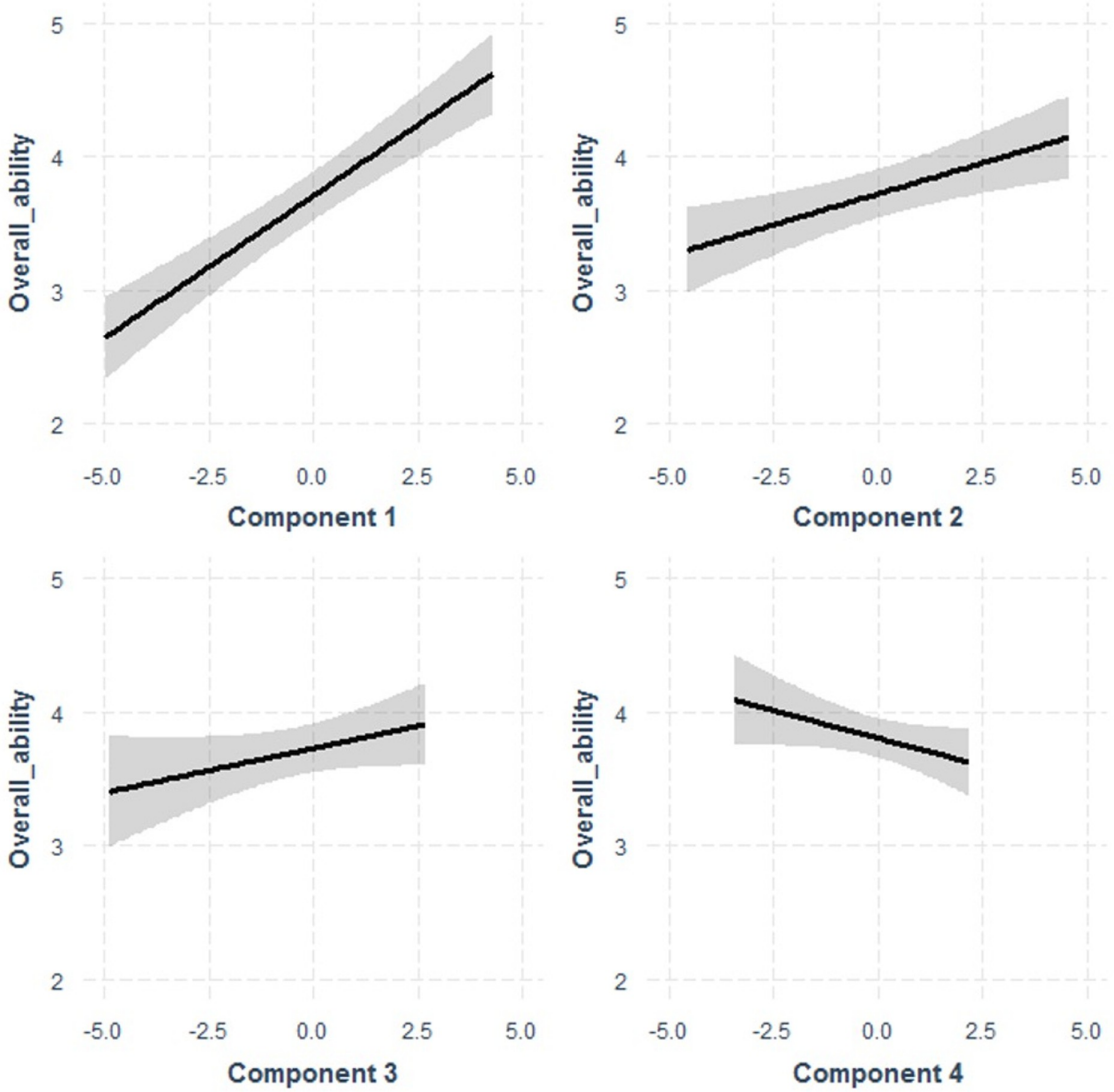
Effect of component on overall ability scores for 210 kelpies as estimated by multivariable mixed model regression. Components 1, 2 and 4 are slopes significantly different from 0.

The effect of each fixed variable on Overall Ability was modelled individually along with the random effect of owner. In univariate modelling, higher PC1 and PC2 were associated with increased overall ability scores, increased PC4 with decreased overall ability score, use of dog for droving with increased overall ability score, and increasing age with improved overall ability score (See Fig 3).

During stepwise deletion was no significant interaction between Age and PC1 (p = 0.301), Age and PC2 (p = 0.307), Age and PC3 (p = 0.551) or Age and PC4 (p = 0.879), leading to the removal of these terms, and suggesting that the relationship between these underlying latent personality traits and perceived overall ability is consistent over the dogs’ working life and also itself not significantly different from zero (b = 0.03, t = 1811, p = 0.07).

Similarly, there was no significant interaction between sex and the removal of gonads (p = 0.261), nor any significant effect of desexing at all (p = 0.820), and so these terms were also removed.

The removal of use in yard work from the model improved the AIC from 355.57 to 354.07 and the removal of paddock work improved the AIC to 353.23. Removal of utility did not improve AIC and droving was significant in the final model. It is possible that utility and droving activities are given to dogs which high perceived overall ability and that yard and paddock work are more fundamental activities.

### Identifying patterns in herding dog personality

The strength of the correlations between personality terms scored for the sample of working Kelpies indicated the internal consistency of the HDAF-P. As highlighted in Table 6, moderate to strong (convergent and divergent) correlations arise between terms expected to measure similar behavioural tendencies, such as the terms confidence, boldness, persistence, timidness and nervousness. In addition, there were some less anticipated convergent correlations between terms, such as calmness and intelligence (T = 0.412, p < 0.001).

**Table 6:** Kendall’s rank correlation co-efficient for a sample of 210 working Kelpies scored using the HDAF-P. Light grey shading indicates significance of raw P<0.05. Dark grey shading indicates significance at P<0.05 after Holm p value adjustment for multiple comparisons.

	Confidence	Calmness	Intelligence	Trainability	Boldness	Patience	Timidness	Persistence	Hyperactivity	Initiative	Excitability	Obedience	Nervousness	Impulsiveness	Sociability	Friendliness	Stamina
Confidence	1.00																
Calmness	0.14	1.00															
Intelligence	0.34	0.41	1.00														
Trainability	0.11	0.34	0.45	1.00													
Boldness	0.59	0.02	0.28	0.13	1.00												
Patience	0.03	0.63	0.40	0.33	-0.04	1.00											
Timidness	-0.40	0.02	-0.14	-0.04	-0.42	0.07	1.00										
Persistence	0.42	0.13	0.27	0.15	0.49	0.16	-0.35	1.00									
Hyperactivity	0.03	-0.51	-0.21	-0.12	0.14	-0.40	-0.02	0.05	1.00								
Initiative	0.39	0.22	0.52	0.27	0.30	0.24	-0.22	0.40	-0.10	1.00							
Excitability	0.03	-0.42	-0.11	-0.03	0.13	-0.37	0.04	0.02	0.69	0.03	1.00						
Obedience	0.07	0.30	0.32	0.62	0.02	0.38	0.09	0.08	-0.13	0.19	-0.07	1.00					
Nervousness	-0.34	-0.20	-0.22	-0.17	-0.27	-0.14	0.49	-0.25	0.18	-0.21	0.16	-0.05	1.00				
Impulsiveness	0.00	-0.32	-0.29	-0.28	0.04	-0.38	0.05	-0.03	0.44	-0.19	0.35	-0.34	0.20	1.00			
Sociability	0.20	0.08	0.15	0.06	0.19	0.03	-0.13	0.07	0.04	0.07	0.05	0.05	-0.16	-0.01	1.00		
Friendliness	0.19	0.13	0.22	0.13	0.21	0.12	-0.09	0.13	0.01	0.09	0.05	0.16	-0.19	-0.07	0.64	1.00	
Stamina	0.22	0.13	0.18	0.18	0.24	0.10	-0.24	0.40	0.02	0.27	0.04	0.07	-0.16	0.06	0.05	0.05	1.00
Overall ability	0.36	0.26	0.32	0.21	0.29	0.23	-0.29	0.36	-0.18	0.41	-0.18	0.10	-0.30	-0.15	0.09	0.08	0.15

Scores for the overall ability of the herding dogs correlated most strongly with the personality terms: initiative (T = 0.41, p < 0.001), persistence (T = 0.36, p < 0.001), intelligence (T = 0.32, p < 0.001), confidence (T = 0.36, p < 0.001), calmness (T = 0.26, p < 0.001) and (inverted) nervousness (T = -0.30, p < 0.001).

## Discussion

Although the HDAF-P was formulated for use on various stock herding dog breeds, narrowing the analysis to a single breed could allow for future use of the data in genomic studies, such as heritability estimations, so the current study focuses on the Australian Working Kelpie.

The HDAF-P appears to have acceptable internal consistency because terms intended to evaluate similar behavioural tendencies align. Significant correlations of above 0.3, in the anticipated direction, supported the expected relationships between terms as articulated in the commonly cited dog personality models. Examples include the correlation of the terms impulsivity and patience, both of which were sourced from the DIAS questionnaire [28] and the terms excitability and hyperactivity which cluster together in the MCPQ-R trait of Extraversion [27].

The bold-shy continuum is considered to represent a spectrum of behaviours from little aversion-to-risk and novelty through to, the antithesis, high stress responsiveness [12, 43]. So, there is an appropriate convergence in the HDAF-P between the terms confidence and boldness which also had divergent correlations with timidness and nervousness. Additional moderate, positive correlations were revealed among boldness traits and the terms initiative and persistence. This relationship aligns with the trait described by Ley et al. [27] as motivation/self-assuredness which contains the terms ‘persevering, independent, tenacious’.

The traits initiative, persistence and confidence were three of the most strongly correlated with the subjects’ overall ability as herding dogs. As these traits are so important in successful stock herding dogs, this result provides further evidence that shyness-boldness predicts success in German Shepherd dogs and Belgian Tervurens, as postulated by Svartberg [43]. However, as Svartberg’s study graded success of dogs over a range of trials that required specialised training such as ‘delivering messages’ and ‘handler protection’, he suggested that boldness leads to success because it predisposes to trainability. In the current study of kelpies, trainability and obedience had only weak positive correlations with confidence and boldness (and a lack of timidness and nervousness), suggesting that boldness influences overall ability for reasons other than rendering dogs trainable. This difference between studies and breeds may not be surprising because successful herding dogs are largely expected to work stock independently and their confidence may trump receptivity to training.

Terms related to behavioural reactivity (hyperactivity, excitability) did not correlate with bold-shy traits. This may reflect a refined understanding among herding dog handlers that a lively, reactive dog may be prone to arousal but still be highly stress-prone (‘panicky’), rather than confident. A negative correlation between hyperactivity/excitability and overall ability is consistent with the Yerkes-Dodson law which describes an ‘inverted U’ relationship between arousal and performance [44]. A logical corollary to the inverse relationship between excitability and overall ability is the current finding of a significant and positive correlation between calmness and overall working ability.

Component 1 of the PCA describes a strong relationship among calmness, patience and intelligence. This relationship seems to confirm the importance of a personality dimension related not simply to motor activity but to emotional reactivity. Evidence of this relationship exists in dogs, humans and other non-human animals [45, 46]. It could be that both a lack of anxiety and a propensity to regulate behaviour facilitate problem-solving in the context of livestock herding. Behavioural regulation and inhibitory control are aspects of impulsivity. Muller et al. [45] found that, depending on the task, inhibitory control had an effect on problem-solving performance. In the current study, a negative correlation between impulsivity and perceived intelligence emerged.

Intelligence in dogs is a complex concept. The term may refer to social cognition, spatial problem-solving ability or speed of learning and memory [47]. Companion animal owners tend to consider their dog’s intelligence level to be synonymous with trainability and obedience [26] and separate from independence. In the current study of herding dogs, moderate positive correlations emerged between intelligence and trainability/obedience. However, the strongest correlation with intelligence was with the term initiative. Initiative is highly suggestive of an ability to assess and act independently which may be quite distinct from simple obedience or compliance. It is perhaps unsurprising that herding dog owners may perceive a difference between obedience (a trait required to excel in competitive livestock working dog trials), and initiative (a trait crucial for paddock dogs mustering large mobs of sheep out of sight of the handler and negotiating obstacles independently).

We hypothesised that impulsivity would not be a trait of value in livestock herding dogs. The results of the correlation analysis were supportive of this in that impulsivity was negatively correlated with overall ability (T = -0.15) and patience was positively correlated with overall ability (T = 0.23).

The emergence of persistence as one of the traits more strongly correlated with overall ability is an interesting in light of a study using selective sweep analysis to look for evidence for the selection pressure placed on the working Kelpie breed [48], as distinct from the Australian bench Kelpie (a non-working, show variety of Kelpie). That study identified, in the working Kelpies, a region with genes related to fear-memory and pain perception. This finding appears highly relevant to traits such as persistence (in the face of fearful or painful environments and circumstances). This attribute may represent the result of intentional breeding strategies or the unintentional evolutionary selection that occurs when some animals simply do not survive (or persist) to breeding age. That said, the current study reveals an awareness, among the current respondents, of the importance of the persistence trait in achieving working success. Thus, it appears that working Kelpie breeders are selecting breeding animals for their behavioural resilience.

The HDAF-P in the current study highlights the behavioural traits owners believe to be most prevalent in herding dogs of perceived high ability. This highlights the traits that should be fostered in breeding, training and husbandry strategies to maximise the ability of the herding Kelpie and reduce the wastage that results from breeding dogs of low working ability.

Future avenues of research should focus on measuring the reliability and validity of the HDAF-P [49, 50]. It is difficult to test the current data for inter-rater reliability of the HDAF-P, because each dog in the current study had only a single handler, but similar scales have shown good inter-rater reliability [15, 28]. Test-retest reliability of the HDAF-P should be measured in future studies by asking owners to repeat score their dogs within a 6-month period [28].

It is expected that the use of industry-generated manuals to source behavioural terminology in the current study has ensured the HDAF-P is relevant and accessible to participants [24]. The decision to use single adjectives, rather than exhaustive definitions, within the HDAF-P (with the exception of impulsivity), as employed by Ley et al. [32], boosted the simplicity and brevity of the method. However, it is recognised that this approach risks compromising standardisation because failing to define the terms and give the rating scale defined criteria for each score may increase the risk of participants differing in their application of the scale, depending on their experience. To assess the consistency of understanding of the terms, there may be merit in a follow-up interviews of dog handlers to record their interpretation of the 17 terms. Owners’ experience with dogs may affect the reliability of their ratings of dogs’ personality [3]. That said, the experience of the current respondents was not assessed. However, given that owners of working dogs are reliant on the dogs for their livelihood, one might imbue the current respondents with more relevant experience than companion dog owners.

External validation ideally requires a comparison of the HDAF-P questionnaire with a so-called gold standard. However, such a standard in herding dog personality assessment is not yet defined [8]. In their validation of a search dog assessment method, Rooney et al. [15] demonstrated that a subjective handler rating method of 13 characteristics using a scale from 1 (low) to 5 (extremely high) corresponded with objective ratings of behaviour in a standardised search task undertaken by 26 dogs. This model cannot be faithfully replicated in a herding test because the behaviour of the herded stock cannot be controlled to standardise such a test. The complexity of the characteristics the herding dog assessment targets for evaluation provide a challenge for measuring external validity. That said, it would be useful to access trial results to determine how well the HDAF-P predicts working ability as assessed by judges, rather than owners. It is worth noting that Patronek and Bradley [51] convincingly argue (albeit in the shelter and rehoming context) that tests to provoke indications of particular behavioural responses may have little value in predicting future outcomes.

The current study found wide ranges of scores for most of the terms in the HDAF-P. This provides the variation required to investigate the genetic contribution to behavioural variation. The trait terms were scored on intensity (very low to very high) rather than desirability (e.g., good or bad). As ideal expression of each trait may vary among working contexts and with handler preference, the current approach attempts to avoid the need to make value judgements. As documented by Arvelius et al. [10], this approach results in improved behavioural assessments. Other variables that we measured regarding specific herding behaviours and dog living conditions have been reported elsewhere [2].

Finally, it is worth noting that the current results pertain to a questionnaire developed for use on the Australian kelpie in Australian contexts. We would not expect the same results for the Australian cattle dog or the Border collie because these breeds are often selected for different traits when interacting with livestock (e.g., Australian cattle dogs are often selected to show more bite and Border collies are not selected for a readiness to travel across the backs of sheep). Furthermore, herding under Australian conditions regularly involves high ambient temperatures and hazards (including prickly vegetation and venomous snakes) and, as such, may differ from herding in other countries.

## Conclusions

The Herding Dog Assessment Form–personality is a brief and easily administered owner questionnaire designed specifically for assessing the personality traits of most relevance to working success in livestock herding dogs. Data collected with this form suggest that the overall ability of stock herding Kelpies is related to these dogs demonstrating behaviours consistent with initiative, persistence, confidence, intelligence and (the inverse of) nervousness.

## Supporting information

S1 File The Herding Dog Assessment Form–personality (HDAF-P). (DOCX)

S2 File RAW DATA with anonymised owner identity. (CSV)
