# Supplementary figures and images for: Valued personality traits in livestock herding Kelpies—Development and application of a livestock herding dog assessment form
Source: PLoS One. 2022 Apr 26;17(4):e0267266. doi: 10.1371/journal.pone.0267266 (PMC9041829; doi:10.1371/journal.pone.0267266)

### Herding Dog Assessment Form – personality component


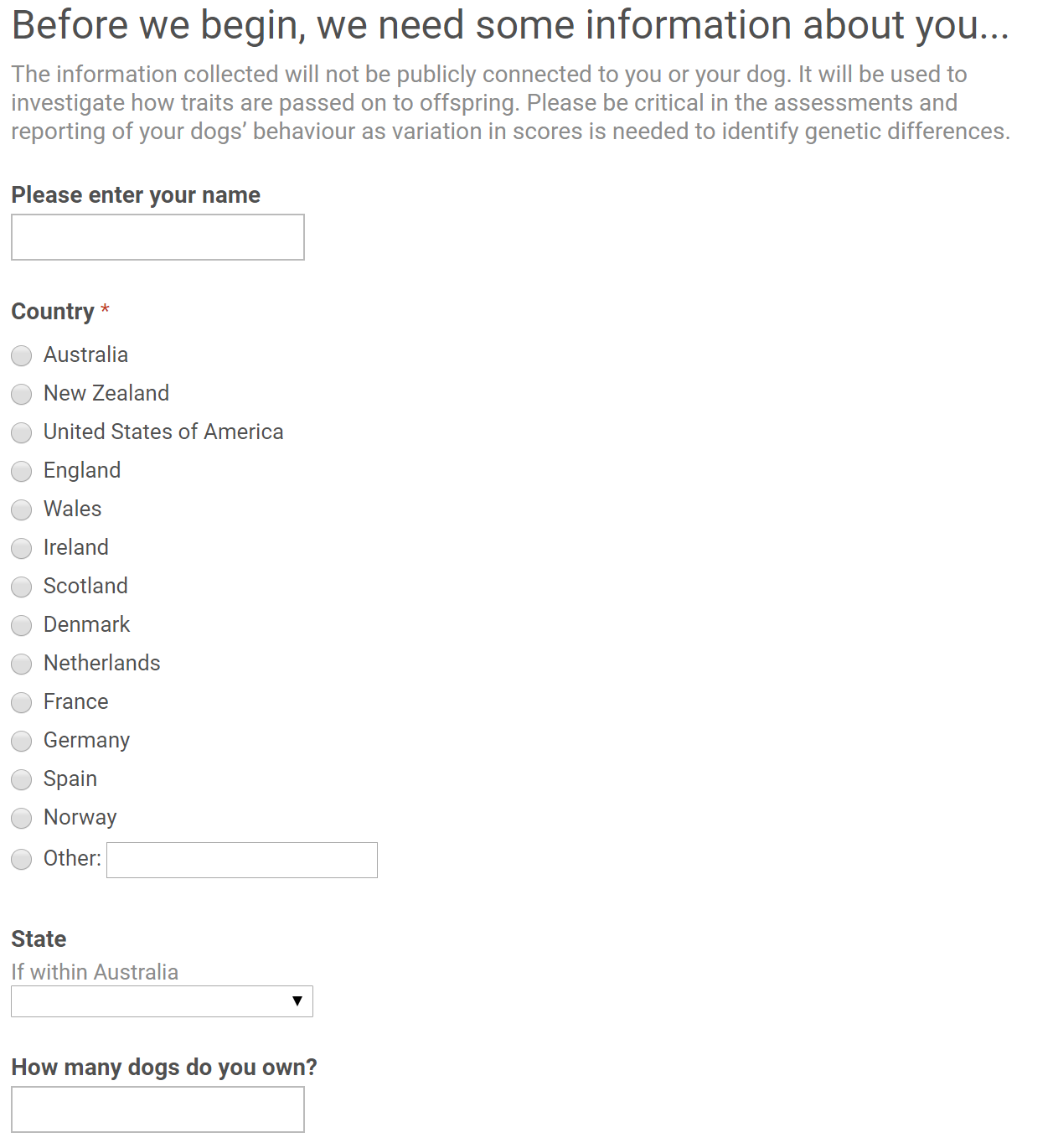


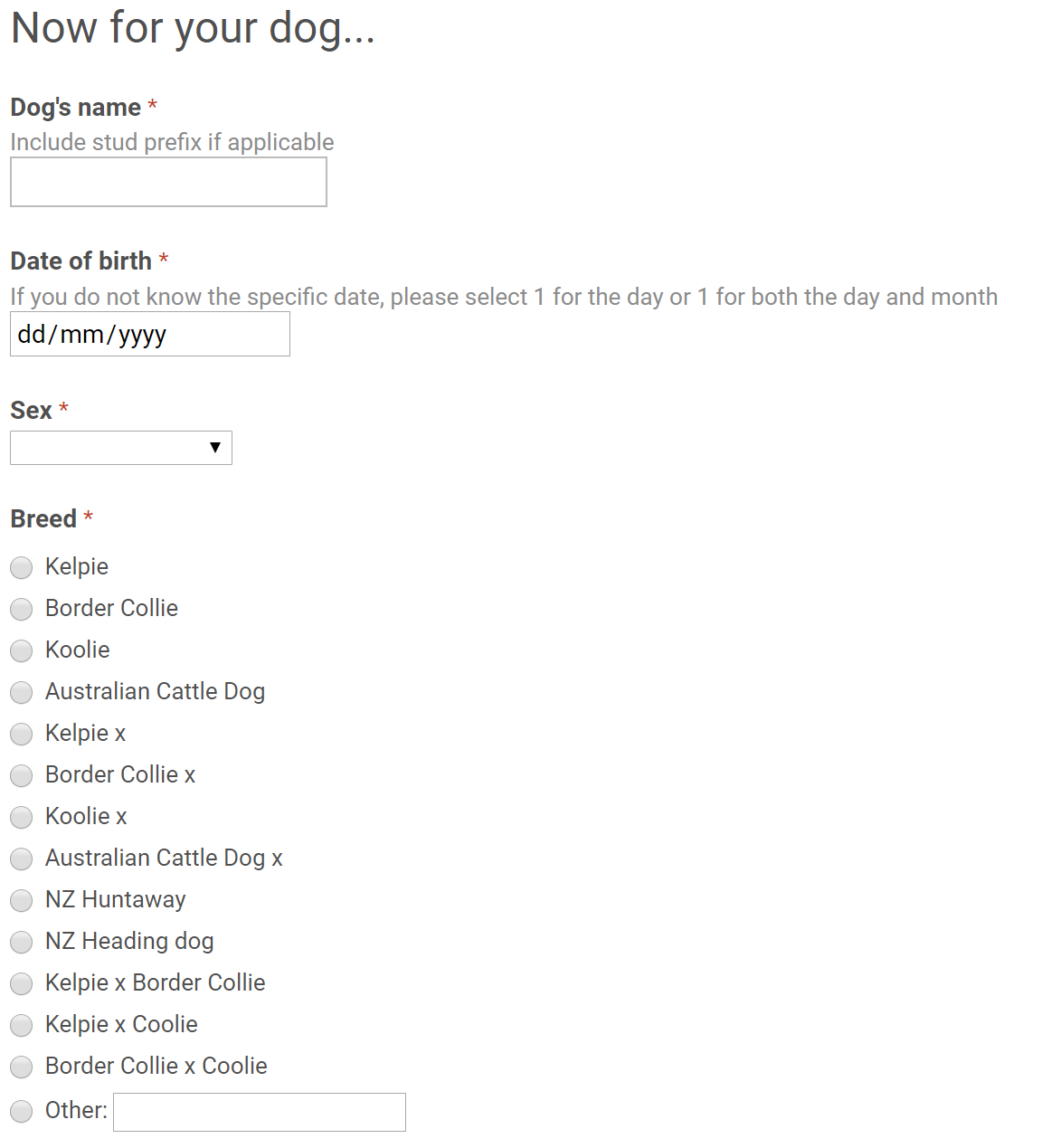

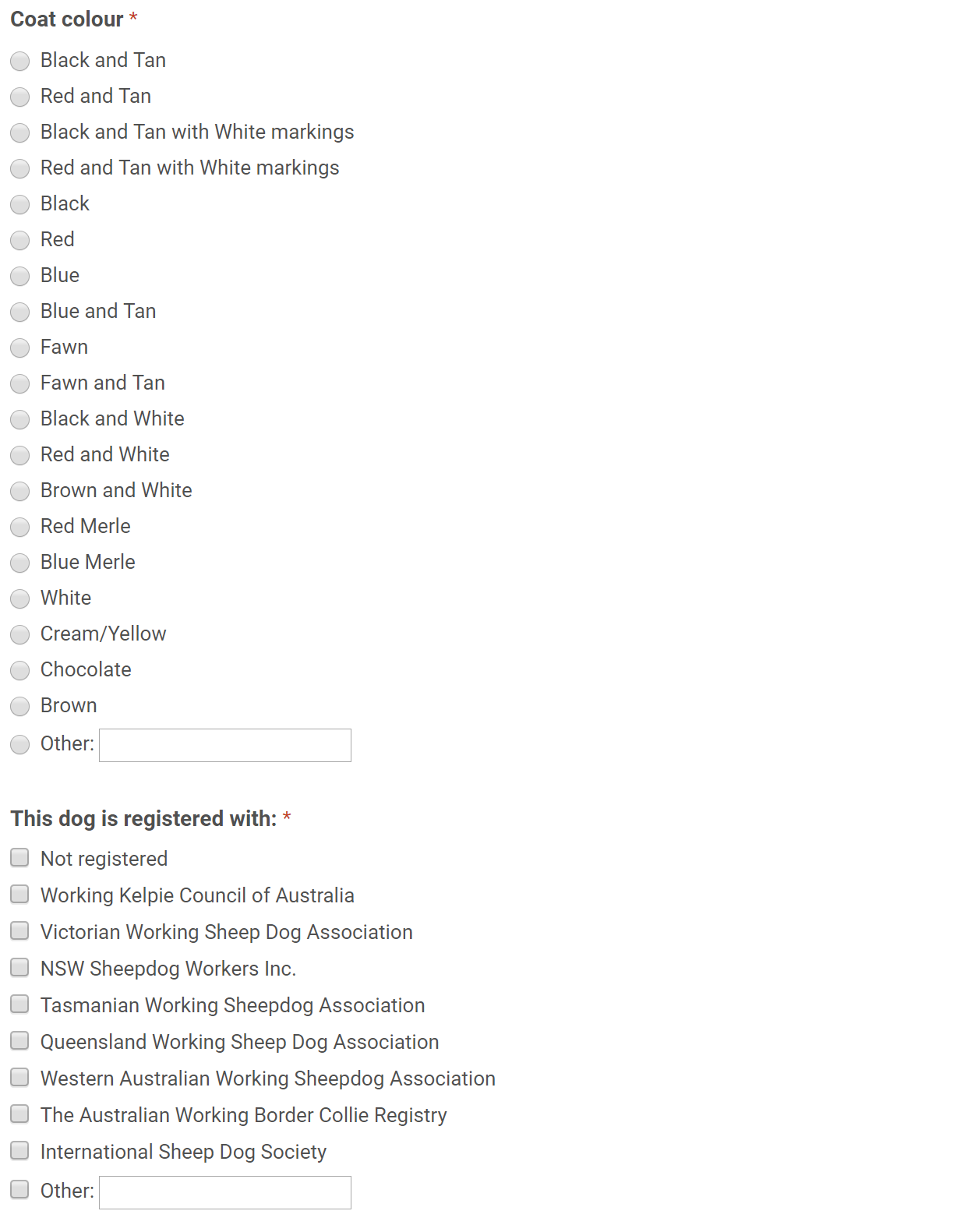

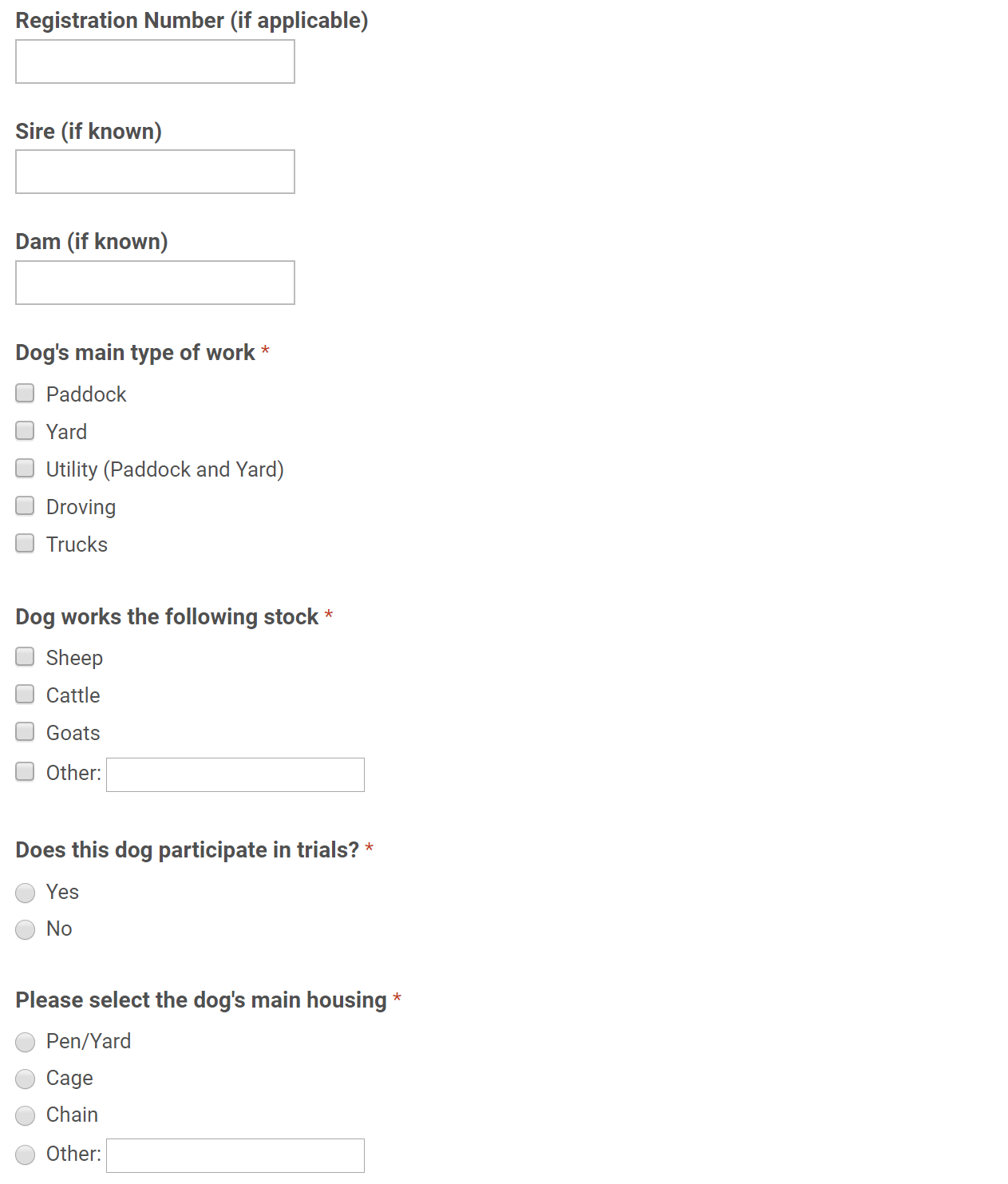

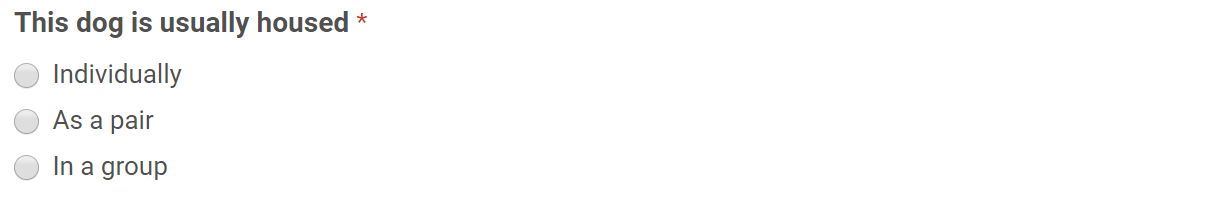

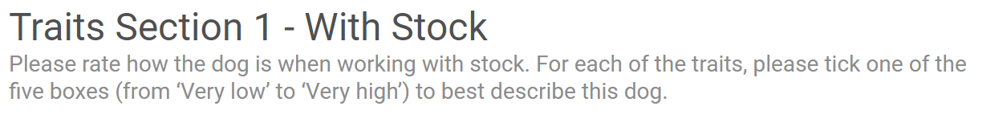

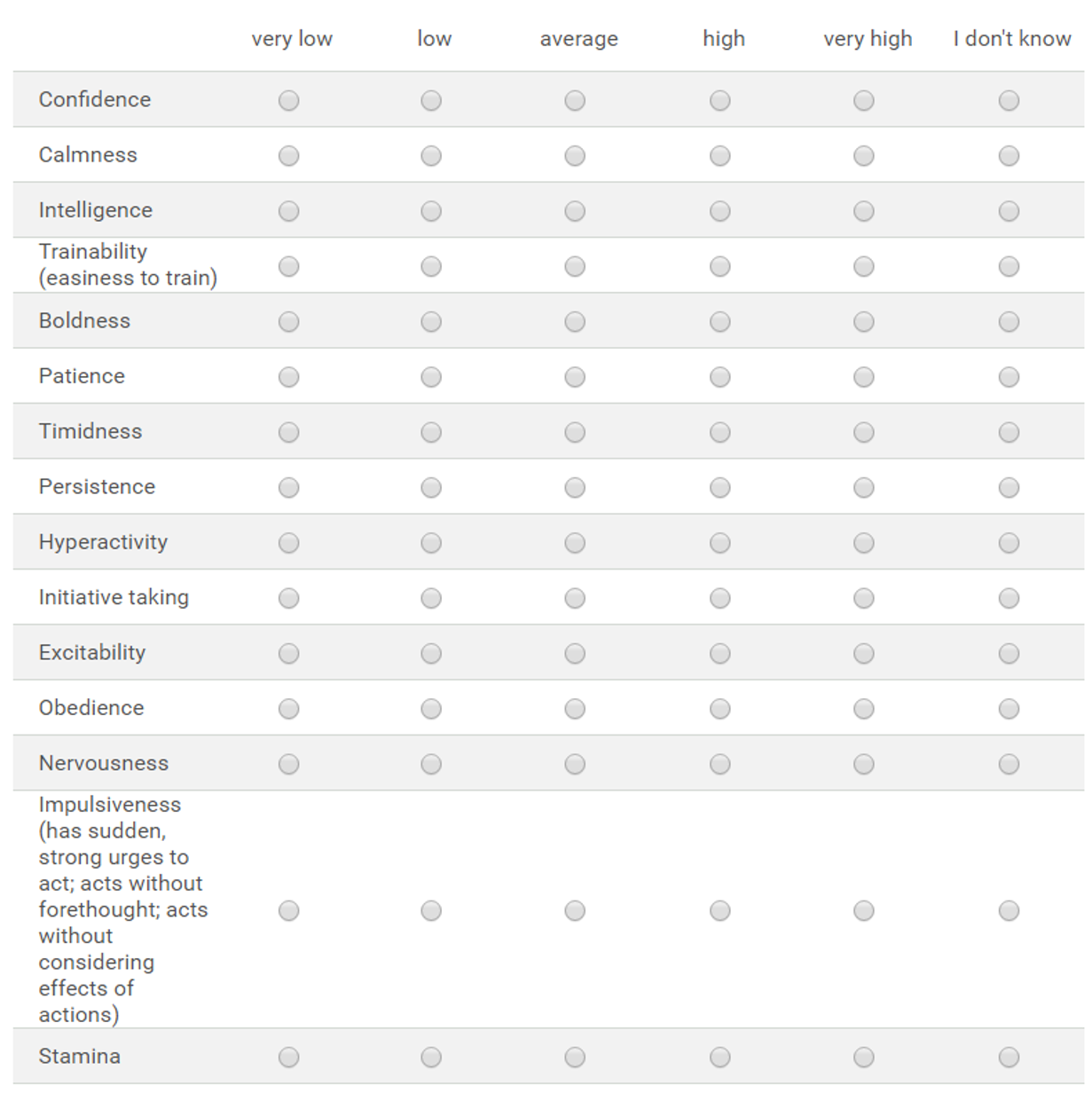

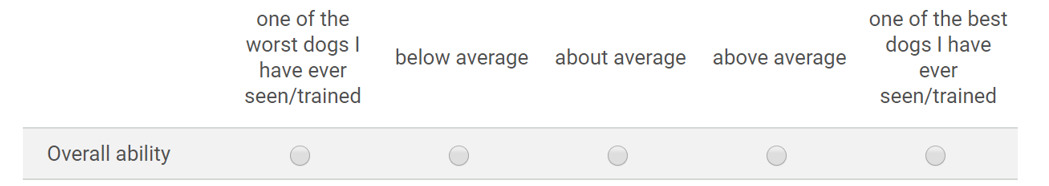

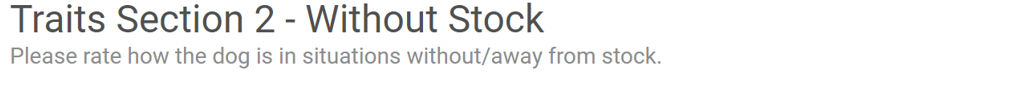


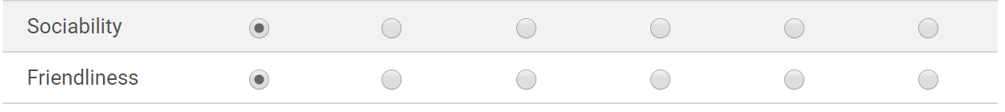

Supplement: S1 File — (DOCX) [file pone.0267266.s001.docx]
